# Inhibition of p38 MAPK Suppresses Inflammatory Cytokine Induction by Etoposide, 5-Fluorouracil, and Doxorubicin without Affecting Tumoricidal Activity

**DOI:** 10.1371/journal.pone.0002355

**Published:** 2008-06-04

**Authors:** Collin R. Elsea, Daniel A. Roberts, Brian J. Druker, Lisa J. Wood

**Affiliations:** 1 School of Nursing, Oregon Health & Science University, Portland, Oregon, United States of America; 2 Howard Hughes Medical Institute, Boston, Massachusetts, United States of America; 3 Division of Hematology and Medical Oncology, Oregon Health & Science University Cancer Institute, Portland, Oregon, United States of America; 4 Department of Radiation Medicine, School of Medicine, Cancer Institute, Oregon Health & Science University, Portland, Oregon, United States of America; Vanderbilt University School of Medicine, United States of America

## Abstract

Cancer patients undergoing treatment with systemic cancer chemotherapy drugs often experience debilitating fatigue similar to sickness behavior, a normal response to infection or tissue damage caused by the production of the inflammatory cytokines IL-1β, TNF-α, and IL-6. The p38 mitogen activated protein kinase (p38 MAPK) plays a central role in the production of these cytokines and consequently the development of sickness behavior. Targeted inhibitors of p38 MAPK can reduce systemic inflammatory cytokine production and the development of sickness behavior. Several systemic cancer chemotherapy drugs have been shown to stimulate inflammatory cytokine production, yet whether this response is related to a common ability to activate p38 MAPK is not known and is the focus of this study. This understanding may present the possibility of using p38 MAPK inhibitors to reduce chemotherapy-induced inflammatory cytokine production and consequently treatment-related fatigue. One caveat of this approach is a potential reduction in chemotherapeutic efficacy as some believe that p38 MAPK activity is required for chemotherapy-induced cytotoxicity of tumor cells. The purpose of this study was to demonstrate proof of principal that p38 MAPK inhibition can block chemotherapy- induced inflammatory cytokine production without inhibiting drug-induced cytotoxicity using murine peritoneal macrophages and Lewis Lung Carcinoma (LLC1) cells as model cell systems. Using these cells we assessed the requirement of etoposide, doxorubicin, 5-flourouracil, and docetaxel for p38 MAPK in inflammatory cytokine production and cytotoxicity. Study findings demonstrate that clinically relevant doses of etoposide, doxorubicin, and 5-FU activated p38 MAPK in both macrophages and LLC1 cells. In contrast, docetaxel failed to activate p38 MAPK in either cell type. Activation of p38 MAPK mediated the drug's effects on inflammatory cytokine production in macrophages but not LLC1 cytotoxicity and this was confirmed with inhibitor studies.

## Introduction

Sickness behavior describes a cluster of symptoms including fatigue, loss of appetite, and disturbed sleep that is initiated by increased production of the inflammatory cytokines IL-1β, TNF-α, and IL-6. Studies in humans and in animal models have demonstrated the role that these cytokines play in the development of sickness behavior [1], [2], [3], [4], [5], [6]. The p38 mitogen activated protein kinase (p38 MAPK) plays a central role in the inflammatory cytokine response to immune challenge and consequently the development of sickness behavior. Specifically, in a recent study a human model of systemic inflammation was used to determine the role of p38 MAPK activity in the cytokine-induced sickness behavior response to low dose (4 ng/kg) bacterial lipopolysaccharide (LPS) [7]. In this model p38 MAPK activity in peripheral blood mononuclear cells (PBMC) peaked within 1-hour of LPS injection, followed by an increase in plasma levels of TNF-α and IL-6 which peaked at 3–4 hours post injection and returned to baseline soon thereafter [7]. The rise in plasma levels of these cytokines coincide with the symptoms of sickness behavior [2], [7]. A similar relationship between p38 MAPK activity and cytokine production was observed *in vitro* using LPS-stimulated PMBCs. To assess the role of p38 MAPK in LPS-induced cytokine production and the induction of sickness behavior, participants were treated with the p38 MAPK inhibitor BIRB796 (Boeringher Ingelheim) prior to LPS injection [7]. BIRB796 pretreatment blocked p38 MAPK activation in PBMC and the rise in plasma cytokine levels in response to LPS injection [7]. Consequently LPS-induced sickness behaviors were attenuated in the BIRB796 pre-treatment group [7]. Similar findings have been obtained from *in vivo* animal studies using a different p38 MAPK inhibitor, SB203580 (Calbiochem) which protected mice from endotoxic shock following administration of a lethal dose of LPS [8].

Over the last decade, there has been much speculation that the fatigue commonly experienced by cancer patients undergoing systemic cytotoxic chemotherapy is the same as sickness behavior (For a recent review see [9]). We propose that the ability of cytotoxic chemotherapy drugs to induce fatigue may be related in part to their ability to induce inflammatory cytokine production via activation of p38 MAPK in target cells. There are two lines of evidence that support this idea. First, cytotoxic chemotherapy drugs have been shown to activate p38 MAPK in several tumor cell lines [10], [11], [12], [13], [14]. Importantly, in this context, p38 MAPK activity has been proposed to play a role in drug-induced cytotoxicity although several studies do not support this [10]. Second, several studies have shown that commonly used cancer chemotherapy drugs can stimulate the production of inflammatory cytokines. Many of these prior studies focused on examining changes in inflammatory cytokines following drug administration in experimental animal models. In this context drug-induced damage to susceptible tissues is likely a significant stimulus for inflammatory cytokine production. We recently found that mice administered etoposide displayed a rapid increase in blood levels of IL-6 that peaked at 3–6 hours post-administration [15]. Similar findings were observed with the alkylating agent cyclophosphamide [16]. Splenocytes collected from mice administered clinically relevant doses of cytarabine, cisplatin, etoposide, or melphalan display an increase in the synthesis of several cytokines, including TNF-α [17]. Moreover, macrophages collected from peritoneal exudates from doxorubicin treated mice displayed increased tumoricidal activity compared to those from untreated mice due most likely to increased production of TNF- α [18]. Finally, cisplatin-induced nephrotoxicity is associated with increased production of TNF- α [19], [20], [21]. In addition to these *in vivo* studies, several *in vitro* studies have shown that cytotoxic chemotherapies can directly stimulate an innate immune response in target cells. Specifically, paclitaxel can induce inflammatory cytokine production in murine macrophage cell lines and in human PBMCs [22], [23], [24], [25], [26], [27], [28], [29] which is most likely related to its ability to mimic the action of bacterial lipopolysaccharide (LPS) [24], [25], [26], [29], [30]. Although paclitaxel can also activate p38 MAPK in these cells it is unclear whether its activity is required for inflammatory cytokine production. Other studies including our own have demonstrated that several other cytotoxic cancer chemotherapy drugs can stimulate the production of inflammatory cytokines in macrophages and monocytes *in vitro*. For instance, we have demonstrated that etoposide can induce IL-6 in murine macrophages *in vitro* [15]. Etoposide has also been shown to induce TNF-α in human monocytes [31]. The active metabolite of cyclophosphamide can induce IL-6 production by murine peritoneal macrophages *in vitro* [16]. In addition, the related alkylating agents cisplatin and carboplatin have also been shown to induce IL-1β, TNF-α and IL-6 production in human monocytes [32] and in murine macrophages [32], [33], [34], [35], [36], [37], [38], [39]. It is important to note that the requirement for p38 MAPK in chemotherapy induced inflammatory cytokine production was not examined in these studies. The requirement for p38 MAPK in this cytokine response was recently investigated by our group. We found that etoposide activated p38 MAPK in murine peritoneal macrophages *in vitro*, and induced the production of IL-6 [15]. Blocking p38 MAPK activity with a specific p38 MAPK inhibitor (ML3403; Calbiochem) blocked etoposide-mediated induction of IL-6 production in these cells [15]. Whether other chemotherapy drugs can induce inflammatory cytokine production via p38 MAPK activation is not known. Understanding whether this is the case may offer the possibility of using p38 MAPK inhibitors to decrease inflammatory cytokine production *in vivo* thereby ameliorating cancer treatment related fatigue. However, one potential caveat to this approach is that p38 MAPK activity is considered by some to be essential for drug induced cytotoxicity. Specifically, several studies employed specific p38 MAPK inhibitors to block the chemotherapy-induced activation of the p38 MAPK pathway. In the majority of these studies, inhibition of p38 MAPK activity in chemotherapy treated cancer cell lines was associated with a modest reduction in drug-induced cytotoxicity [14]. However, an important limitation of these studies is that they fail to establish a direct correlation between the activation of p38 MAPK by cancer chemotherapy drugs and drug-induced cytotoxicity.

The purpose of this study was to demonstrate proof of principal that p38 MAPK inhibition can block cytotoxic chemotherapy-induced inflammatory cytokine production without inhibiting drug-induced cytotoxicity using murine peritoneal macrophages and Lewis Lung Carcinoma (LLC1) cells as model cell systems. Using these cells we assessed the requirement of etoposide, doxorubicin, 5-flourouracil, and docetaxel for p38 MAPK in inflammatory cytokine production and cytotoxicity. These drugs were chosen because they are commonly used in the treatment of cancer and represent different drug categories: epidodophyllotoxins (etoposide), anthracyclines (doxorubicin), anti-metabolites (5-FU), and mitotic inhibitors (docetaxel). Study findings demonstrate that clinically relevant doses of etoposide, doxorubicin, and 5-FU activated p38 MAPK in both macrophages and LLC1 cells. In contrast docetaxel at the dosage used failed to activate p38 MAPK in either cell type. Activation of p38 MAPK mediated the drug's effects on inflammatory cytokine production in macrophages but not LCC1 cell cytotoxicity and this was confirmed with inhibitor studies.

## Results

### The induction of inflammatory cytokines by etoposide, 5-FU, doxorubicin, and docetaxel in murine macrophages *in vitro* is mediated by p38 MAPK

**Figure 1:**
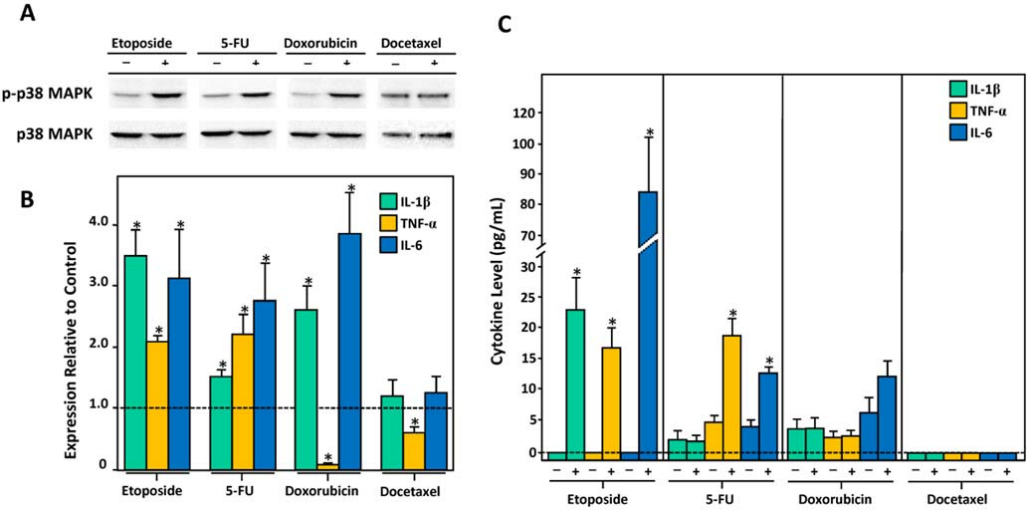
The induction of inflammatory cytokines by etoposide, 5-FU, doxorubicin, and docetaxel in murine macrophages *in vitro* is mediated by p38 MAPK. A) Levels of phosphorylated p38 MAPK and total p38 MAPK in murine peritoneal macrophages incubated with etoposide, 5-FU, doxorubicin, and docetaxel were assessed by immunoblotting. B) Relative levels of IL-1β, TNF-α and IL-6 mRNA in etoposide, 5-FU, doxorubicin, and docetaxel-treated macrophages assessed by QRT-PCR. Fold increase in expression of each cytokine mRNA in drug-treated cells relative to carrier-treated cells is shown. C) Levels of IL-1β, TNF-α and IL-6 in the culture medium of macrophages incubated with etoposide, 5-FU, doxorubicin, and docetaxel.

**Figure 2:**
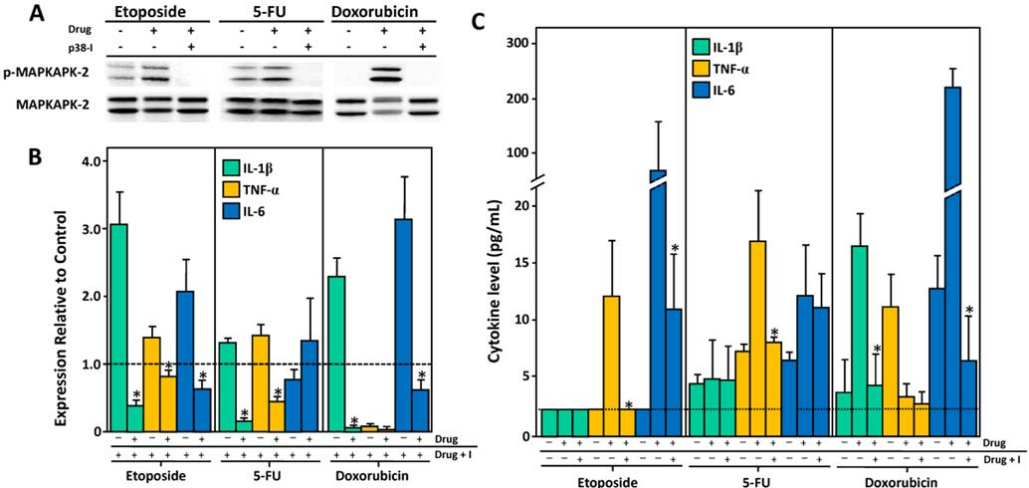
The effect of p38 MAPK blockade on etoposide, 5-FU, and doxorubicin mediated induction of inflammatory cytokine production in murine macrophages. (A) Inhibition of p38 MAPK leads to a reduction in MAPKAPK-2 phosphorylation , a downstream target of p38 MAPK. (B) The effect of pre-treatment with the p38 MAPK inhibitor on IL-1β, TNF-α and IL-6 mRNA levels. Fold increase in expression of each cytokine mRNA in drug treated cells and in cells treated with drug plus inhibitor relative to carrier treated cells is shown. (C) Levels of IL-1β, TNF-α and IL-6 in the culture supernatant of drug treated cells. A significant decrease (p<0.05) in cytokine level in cells treated with drug plus p38 MAPK inhibitor relative to cells treated with drug alone is denoted by an asterisk.

In our earlier study we showed that etoposide could activate p38 MAPK in murine macrophages [15]. To determine whether other cytotoxic chemotherapeutic drugs from different drug classes share a common ability to activate p38 MAPK in these cells, we incubated macrophages with clinically relevant concentrations of etoposide (50 µM), 5-fluorouracil (250 µM), doxorubicin (1 µM), and docetaxel (5 µM). We found that similar to etoposide, 5-FU and doxorubicin could also activate p38 MAPK, as evidenced by an increase in phosphorylated p38 MAPK relative to total p38 MAPK in drug-treated versus carrier-treated cells (Figure 1A). In contrast docetaxel failed to activate p38 MAPK at a dose of 5 µM (Figure 1A) and this finding was observed even when cells were treated with supraphysiological doses (100 µM) of the drug (Data not shown). Consistent with a role for p38 MAPK activation in inflammatory cytokine production we found that drug-induced p38 MAPK activity was associated with increased production of IL-1β, TNF-α, and IL-6. Specifically, etoposide, 5-FU, and doxorubicin treatment led to a significant accumulation of IL-1β and IL-6 mRNAs (Figure 1B). TNF-α mRNA levels also increased in etoposide and 5-FU-treated cells but decreased in those treated with doxorubicin (Figure 1B). A similar decline in TNF-α mRNA was observed in cells treated with docetaxel (Figure 1B). In general, increased levels of cytokine mRNAs were associated with an increase in the corresponding cytokine in the culture medium (Figure 1C). The exceptions were IL-1β in 5-FU-treated cells and all three cytokines in doxorubicin-treated cells. In the case of doxorubicin we found that prolonged exposure of cells to doxorubicin (>24 hr) lead to significant cell death which most likely explained why levels of IL-1β, TNF-α and IL-6 did not accumulate above the threshold of detection by immunoassay in the culture medium. We found that by increasing the concentration of doxorubicin to 10 µM levels of cytokines were detectable and reflected the levels of cytokine mRNA (data not shown and Figure 2).

To determine whether p38 MAPK activity was required for chemotherapy-induced inflammatory cytokine production we pre-treated cells with the p38 MAPK inhibitor prior to the addition of 50 µM etoposide, 250 µM 5-FU, or 10 µM doxorubicin. Pre-treatment with the p38 MAPK inhibitor was sufficient in blocking p38 MAPK signaling as evidenced by a decrease in levels of phosphorylated- MAPKAPK-2, a downstream target of activated p38 MAPK (Figure 2A).

Suppression of p38 MAPK activity in etoposide, 5-FU, and doxorubicin-treated cells blocked the accumulation of one or more of the cytokines (Figure 2B & C). DMSO, the p38 MAPK inhibitor carrier, appeared to decrease the magnitude of the IL-1β, TNF-α, and IL-6 response to etoposide, 5-FU, and doxorubicin treatment (Compare Figures 1B & C and Figure 2 B & C). Nonetheless, these experiments demonstrate that the induction of inflammatory cytokines by etoposide, 5-FU, and doxorubicin in murine macrophages is p38 MAPK dependent.

### The cytotoxic effects of etoposide, 5-fluorouracil, doxorubicin, and docetaxel are not associated with p38 MAPK activation in LLC11 cells

**Figure 3:**
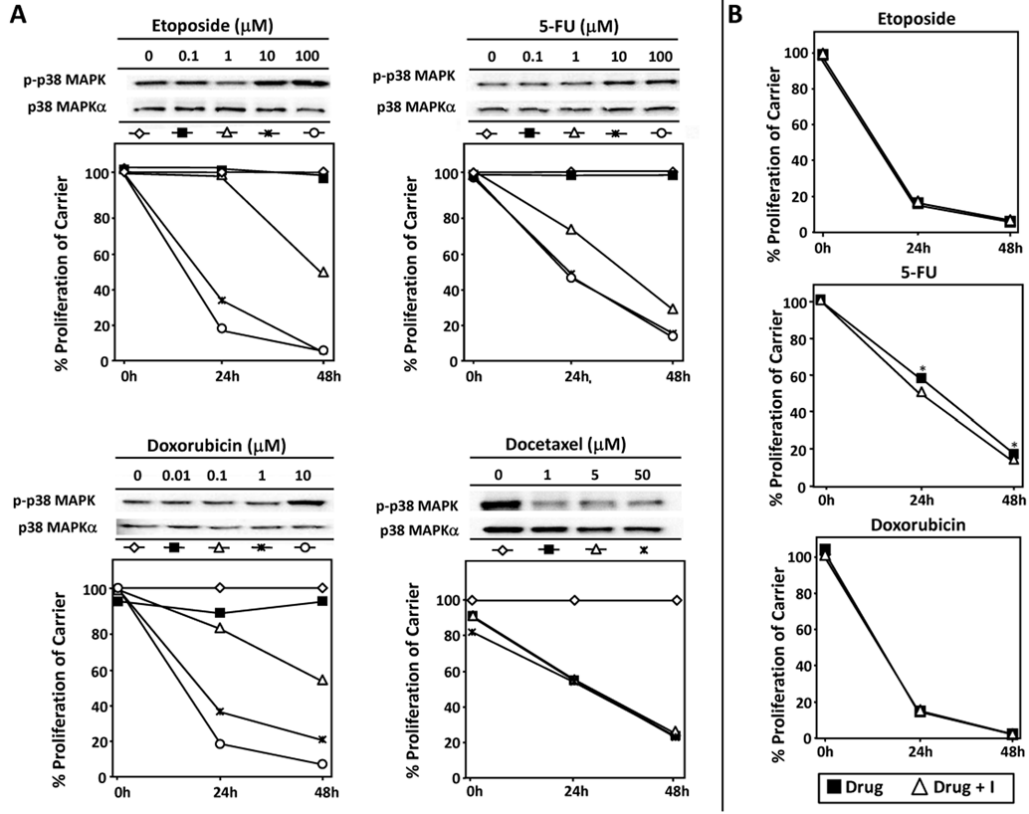
The cytotoxic effects of etoposide, 5-fluorouracil, doxorubicin, and docetaxel are not associated with p38 MAPK activation in LLC11 cells. A) The effect of increasing concentrations of etoposide, 5-FU, doxorubicin or docetaxel on p38 MAPK activation and proliferation were assessed by immunoblotting and MTS assay respectively. B) The effect of p38 MAPK blockade on drug-induced cytotoxicity in LLC1 cells treated with etoposide, 5-FU or doxorubicin. Note that the addition of the p38 MAPK inhibitor appeared to enhance the cytotoxic effects of 5-FU on LLC1 cells (see *).

Previous studies have implicated p38 MAPK activation in chemotherapy-induced cytotoxicity. One limitation of these earlier studies is that they used supraphysiological doses of drug and failed to establish a direct correlation between the activation of p38 MAPK and drug-induced cytotoxicity. To address these limitations we performed proliferation assays with increasing concentrations of each drug. Etoposide, 5-FU, doxocrubicin, and docetaxel were able to inhibit the proliferation of LLC11, although we did not observe a direct relationship between p38 MAPK activation and cytotoxicity (Figure 3A). Indeed, docetaxel decreased p38 MAPK activity in LLC1 cells. These findings suggest that p38 MAPK is not a pre-requisite for chemotherapy-induced cytotoxicity in LLC1 cells. Nonetheless at higher concentrations of etoposide (100 µM), doxorubicin (10 µM), and 5-FU (100 µM) p38 MAPK was evident (Figure 3 A). Therefore to confirm that p38 MAPK activation was not required for drug-induced cytotoxicity we performed proliferation assays with these drug concentrations in the presence or absence of the p38 MAPK inhibitor. Blockade of p38 MAPK activity did not inhibit the anti-proliferative effects of etoposide, 5-FU and doxorubicin (Figure 3 B). In fact, p38 blockade enhanced the anti-proliferative effects of 5-FU (Figure 3B). Taken together our data show that some cancer chemotherapy drugs can activate the p38 MAPK pathway, but the biological consequences of its activation in LLC1 remain unclear.

## Discussion

It has long been speculated that the fatigue that occurs in cancer patients during treatment with systemic cytotoxic chemotherapy drugs may be caused by an increase in production of inflammatory cytokines and thereby could be similar to sickness behavior. Several studies demonstrating the induction of inflammatory cytokines in response to cytotoxic chemotherapies in immunologically-derived cells *in vitro* and in experimental animals support this idea. The p38 MAPK plays an important role in the production of inflammatory cytokines and the development of sickness behavior and activation of p38 MAPK has been observed in several tumor cell lines treated with a variety of cytotoxic chemotherapies. Targeted inhibitors of p38 MAPK can decrease the inflammatory cytokine response to immune challenge and decrease sickness behavior and several pharmaceutical companies are currently investing in the development of p38 MAPK inhibitors for the treatment of inflammatory diseases [40], [41], [42]. Understanding whether p38 MAPK plays a role in the induction of inflammatory cytokines in response to cytotoxic chemotherapies, could support the use of p38 MAPK inhibitors in the management of cancer treatment-related fatigue. However, there have been few studies that have assessed whether the inflammatory cytokine response to chemotherapy occurs in a p38 MAPK-dependent manner. The data presented herein show a direct role for p38 MAPK in the induction of inflammatory cytokines by etoposide, 5-FU, and doxorubicin using murine macrophages as a model system.

Not all of the chemotherapy drugs tested in this study were able to activate p38 MAPK; docetaxel failed to activate p38 MAPK in either macrophages or LLC1 cells even at supraphysiological concentrations. These findings are consistent with those of a prior study in which 100 µM docetaxel failed to induce inflammatory cytokines in the murine macrophage cell line RAW 246.7 and in peritoneal macrophages collected from LPS-responsive CH3/OuJ mice [25]. In these earlier studies the ability of docetaxel to activate p38 MAPK was not investigated. Our findings suggest that its failure to induce inflammatory cytokine production in immune cells is most likely due to its failure to activate p38 MAPK. In light of this finding and for our hypothesis to be correct we would expect that docetaxel will not cause sickness behavior (i.e. fatigue) because it does not activate p38 MAPK and consequently fails to induce inflammatory cytokine production in target cells. Unfortunately there are no studies in cancer patients that have focused on determining whether certain drug types are associated with fatigue more than others. Indeed, testing this hypothesis in the clinical setting may be difficult as docetaxel is often administered in combination with other chemotherapeutics making the contribution of this drug alone to fatigue burden impossible to determine. An alternative approach would be to examine the inflammatory cytokine response to docetaxel administration alone in a mouse model of sickness behavior. We used a similar approach recently to determine whether etoposide could induce inflammatory cytokine production and sickness behaviors when administered to mice [15]. Using this model we would expect that a clinically relevant dose of docetaxel would not induce inflammatory cytokine production or sickness behavior. These experiments are clearly warranted and are currently underway.

Of interest was the finding that doxorubicin and docetaxel decreased production of TNF-α in macrophages. Our findings are consistent with previous studies in which doxorubicin failed to induce the production of TNF-α in peritoneal exudates cells from doxorubicin treated mice [37]. Inhibition of p38 MAPK activity in doxorubicin-treated cells failed to further decrease TNF-α production by these cells. Taken together these findings suggest that the ability of doxorubicin and docetaxel to decrease TNF-α level in macrophages is p38 MAPK independent. The fact that decreased TNF-α levels can occur in the context of increased production of IL-1β and IL-6 suggests that depending upon the stimulus, IL-1β, TNF-α and IL-6 production by murine macrophages are governed by distinct molecular events.

Why docetaxel fails to activate p38 MAPK may be related to its ability to induce oxidative stress [43]. Indeed unlike docetaxel, both etoposide and doxorubicin are known to be strong inducers of reactive oxygen species (ROS). In the case of doxorubicin, ROS induction is believed to mediate the cardiotoxic effects of this drug [44]. Although, 5-FU known to be a weak inducer of ROS, like docetaxel, it still induced p38 MAPK in both macrophages and LLC1 in the present study. Our experiments using DMSO, the p38 MAPK inhibitor carrier, also implicate oxidative stress at least in part in drug-induced p38 MAPK activation. Specifically DMSO appeared to decrease the magnitude of the IL-1β, TNF-α, and IL-6 response to etoposide, 5-FU, and doxorubicin treatment (Compare Figures 1B & C and Figure 2 B & C). Clearly additional experiments are needed to understand the relationship between drug-induced oxidative stress, and p38 MAPK activation in these cell types. Understanding whether drug-induced oxidative stress underlies p38 MAPK activation and inflammatory cytokine production in this context may provide an additional strategy to ameliorate fatigue in cancer patients undergoing treatment with certain cancer chemotherapeutics. However one important consideration is the effect of p38 MAPK blockade on drug-induced cytotoxicity. For several tumor cell lines, *in vitro* activation of p38 MAPK following exposure to mechanistically different chemotherapeutic agents has been established [45], [46], [47]. Our data further support the ability of some cytotoxic chemotherapies to activate p38 MAPK in LLC1 cells. However, our findings fail to support the idea that p38 MAPK activity is essential for drug-induced cytotoxicity. First we did not examine a direct correlation between drug-induced inhibition of LLC1 cell proliferation and p38 MAPK activation. Although high concentrations of etoposide, 5-FU and doxorubicin did activate p38 MAPK, further experiments with a p38 MAPK inhibitor did not support a role for this enzyme in drug induced cell killing. In the case of 5-FU, pre-treatment of cells with the p38 MAPK inhibitor enhanced the cytotoxic effects of this drug. Boldt et al., also demonstrated a similar enhancement of the cytotoxic effects of etoposide in several tumor cell lines by the p38 MAPK inhibitor SB203580 [47]. Our data support the idea that p38 MAPK blockade may selectively diminish cytotoxic chemotherapy drug-induced inflammatory cytokine production without decreasing their anti-tumorigenic activity. Clearly additional studies are warranted to assess the consequence of p38 MAPK blockade on tumor growth and progression and treatment related sickness behaviors in *in vivo* studies. While these experiments are currently underway there are other studies that suggest the feasibility of this approach. For instance, p38 MAPK has been shown to play an important role in cisplatin-induced nephrotoxicity in mice most likely through its ability to induce the production of TNF-α [19]. Pre-treatment of mice with the p38 MAPK inhibitor SB203580 significantly reduced cisplatin-induced nephrotoxicity [19]. It is important to note that the effects of p38 MAPK inhibition on the anti-tumor effects of cisplatin were not examined. However, a similar study using an inhibitor of oxidative stress decreased cisplatin-induced cytotoxicity while having no effect on tumor growth [48]. Additional studies in this field may lead to new treatment strategies aimed at reducing fatigue in cancer patients undergoing systemic chemotherapy.

## Materials and Methods

### Reagents

Etoposide (20 mg/ml) solution was purchased from Novation (Irving, TX, USA). Etoposide carrier (2 mg/ml citric acid, 30 mg/ml benzyl alcohol, 80 mg/ml Tween-80, 650 mg/ml polyethylene glycol 400, and 30.5% (v/v) ethanol) was used as an experimental control. Doxorubicin-HCL was purchased as a lyophilized 10 mg tablet from Bedford Labs (Bedford, OH, USA) and was dissolved in sterile, deionized water to attain a stock solution of 1 mg/ml, stored at 4°C. 5-FU was purchased as a 50 mg/ml solution from American Pharmaceutical Partners (Schaumburg, IL, USA). Sterile, deionized water served as a carrier control for doxorubicin and 5-FU in experiments. Docetaxel powder was purchased from Sigma Aldrich, Inc. (Dallas, TX, USA) and stored as a 25 mg/ml solution in dimethylsulfoxide (DMSO) at 4°C. ML3403 (p38 MAPK Inhibitor III) was purchased from CalBiochem (San Diego, CA, USA) and was prepared as a 10 mM stock solution in DMSO. RPMI 1640 cell culture media was purchased from Gibco (Grand Island, NY, USA). Fetal bovine serum (FBS) was purchased from Atlanta Biologicals (Lawrenceville, GA, USA) and was heat-inactivated by 30 min incubation at 56°C in a water bath, followed by 30 min in an ice bath, prior to use in cell culture. Brewer thioglycolate broth was obtained from Sigma-Aldrich, Inc. and was prepared as an 4% (w/v) aqueous solution, aged in the dark for at least one month prior to use.

### Mice

Female 8-week-old C57BL/6 mice purchased from Jackson Laboratories (Bar Harbor, ME) were housed five to a cage in pathogen-free rooms (12-hour light-dark cycle) and had ad lib access to food and water. At the time of sacrifice, mice were terminally sedated using isofluorane according to protocols established at OHSU Department of Comparative Medicine.

### Murine Macrophage Harvest, Culture, and Drug Treatment

Mice were administered 1 cc of sterile and aged 4% Brewer thioglycolate broth by intraperitoneal (IP) injection. Thioglycolate-elicited macrophages were harvested 3 days later by IP lavage with 10 cc sterile, room temperature Dulbecco's phosphate buffered saline (DPBS). Cells were seeded at 4×10^6^ viable cells per 6 cm dish in 3 mL RPMI culture media containing 2% heat-inactivated serum, 100 U/ml penicillin, and 100 µg/ml streptomycin (RPMI-2). After 24-hours of incubation at 37°C/5% CO_2_, non-adherent cells were washed from the wells with DPBS and etoposide, doxorubicin, docetaxel or 5-fluorouracil was added to select wells to final concentrations of 50 µM, 1 µM or 10 µM, 5 µM and 250 µM, respectively, in final volumes of 1.5 ml of culture medium. The drug concentrations used reflect peak plasma concentrations observed during infusion in cancer patients [49], [50], [51], [52]. Separate wells were also incubated with drug carrier alone. Each cell treatment was set up in duplicate, for harvest at 6 & 48 hrs. In p38 MAPK inhibitor studies macrophages were pretreated at 37°C/5% CO_2_ for 1 hour with 1.4 ml RPMI-2 containing 0.1% DMSO or 1.4 ml of RPMI-2 containing 10 µM ML3403. Following one hour of pretreatment, 50 µM etoposide, 250 µM 5-fluorouracil, or 10 µM doxorubicin, were added to select wells, in final volumes of 1.5 ml. The drug concentrations used reflect peak plasma concentrations observed during infusion in cancer patients [49], [50], [51], [52]. Separate wells were also incubated with drug carrier alone. Each cell treatment was set up in duplicate, for harvest at 6 & 48 hrs. After 6 & 48 hrs incubation at 37°C/5% CO_2_, cell culture media was harvested and centrifuged at maximum speed in a micro centrifuge for 3 min to remove any cells. Residual culture media was removed from cells by vacuum, and macrophage monolayers were disrupted using 500 µl buffer RLT (Qiagen, Valencia, CA, USA) containing 10 µl β-mercaptoethanol per ml with the aid of sterile cell lifters. Lysates were homogenized on QiaShredder columns (Qiagen, Valencia, CA, USA) according to manufacturer protocol. Cell lysates and cell-free culture media were stored at −70°C until use. Each experiment was repeated in at least triplicate for all drug combinations and time points.

### Macrophage RNA and Protein Extraction

Total cellular RNA and protein were extracted concurrently from macrophage lysates in buffer RLT using a method described previously [53]. Briefly, RNA was extracted from cell lysates on RNeasy Mini Spin Columns (Qiagen, Valencia, CA, USA) according to the manufacturer's protocol for animal cells. The optional, on-column RNase-free DNase treatment was included to remove any contaminating genomic DNA. Column effluent was saved, pooled for each sample, and stored at <−20°C for 24–48 hrs to allow protein precipitation. Precipitated protein was collected by centrifugation at >10,000 g and 4°C, washed 3× in −20°C 100% ethanol, dried at room temperature, and then dissolved/denatured in SDS-PAGE loading buffer prior to western blot analysis. RNA was used for qRT-PCR measurements of relative IL-1β, TNF-α, and IL-6 mRNA levels.

### Bead-based Immunoflourescence Assay

Levels of IL-1β, TNF-α, and IL-6 in macrophage culture media were measured in duplicate in 3 separate assays using a bead-based immunoflourescence assay (Luminex Inc., Austin, TX, USA). Multiple cytokine analysis kits (LINCOplex kits) were obtained from Linco Research Inc. (St. Charles, MO, USA) and assays were performed according to the protocol supplied by the manufacturer. Data were collected and analyzed using the Luminex-100 system Version IS (Luminex, Austin, TX, USA). A four or five-parameter regression formula was used to calculate the sample concentrations from the standard curves.

### Lewis Lung Cancer Cell Culture, Drug Treatment & Proliferation Assays

Lewis Lung Carcinoma 1 (LLC1) cells were previously derived from a spontaneous, epidermoid lung carcinoma isolated from a C57 Black/6 mouse [54], and were purchased from the American Type Culture Collection (Atlanta, GA, USA). LLC1 cells were cultured in DMEM supplemented with 10% FBS, 100-U/ml penicillin, and 100 µg/ml streptomycin at 37°C in a humidified incubator supplied to 5% of the atmosphere with CO_2_. To determine whether etoposide, 5-FU doxorubicin and docetaxel could activate p38 MAPK, cells were plated onto 100 mm tissue culture dishes and incubated with increasing concentrations of each drug. At 5 hours post drug addition, cells were washed, and protein lysates prepared for immunoblotting. For proliferation assays cells were plated at 4×10^3^ cells per well in 100 µl on 96-well plates. Following incubation at 37°C 5% CO_2_ overnight, etoposide, 5-FU, doxorubicin, and docetaxel were added to the cells at concentrations described in Figure 3. At 0, 24, and 48 h post-addition of chemotherapeutic, cell viabilities in cultures were determined by addition of 3-(4,5-dimethylthiazol-2-yl)-5-(3-carboxymethoxyphenyl)-2-(4-sulfophenyl)-2H-tetrazolium (MTS) cell proliferation reagent, incubated for 3 h at 37°C and 5% CO_2_, and subsequently read at an absorbance at 490 nm. Seven wells of culture were allotted for each drug concentration for each time point. To evaluate the effect of p38 MAPK inhibition on the ability of 50 µM etoposide, 100 µM 5-FU, and 10 µM doxorubicin to inhibit LLC1 cell proliferation, cells were plated as described above and then pre-treated with ML3403 at 10 µM or with DMSO carrier for 1 h at 37°C and 5% CO_2_. The final concentration of DMSO in each culture was 0.1% (v/v). Following p38 MAPK inhibitor pre-treatment, chemotherapeutic drugs were added, followed by continued incubation at 37°C and 5% CO_2_. At 0, 24, and 48 h post-addition of chemotherapeutic MTS assays were performed as described above. All assays were performed in triplicate.

### Immunoblotting

Protein lysates from macrophages or LLC1 cells were resolved by 4–15% gradient SDS-PAGE followed by transfer to polyvinylidene fluoride (PVDF) membranes. PVDF membranes were blocked in a solution of 5% (w/v) bovine serum albumin, 10 mM tris-HCL (pH8.0), 150 mM NaCl, 0.05% (v/v) Tween-20. Immunoblotting was performed by gently rocking overnight at 4°C in blocking solution supplemented with one of the following antibodies: rabbit anti-phospho-p38 MAPK (Thr180/Tyr182) polyclonal antibody (1∶1000, #9211, Cell Signaling Technology); rabbit anti-p38 MAPKα polyclonal antibody (1∶1000, #9218, Cell Signaling Technology); rabbit anti-phospho-MAPKAPK-2 (Thr334) monoclonal antibody (1∶1000, #3007, Cell Signaling Technology); rabbit anti-MAPKAPK-2 polyclonal antibody (1∶1000, #3042, Cell Signaling Technology). Primary antibody binding was detected with goat anti-rabbit IgG-horseradish peroxidase (HRP) conjugate (1∶5000, W401B, Promega Corp., Fitchburg, WI) and visualized using enhanced chemiluminescence (ECL; Pierce, Rockford, IL, USA). ECL was recorded and quantified using a Lumi-Imager in conjunction with LumiAnalyst software (Boehringer Mannheim, Roche Diagnostics, Indianapolis, IN, USA).

### Quantitative Real Time Polymerase Chain Reaction (qRT-PCR) Analysis

Relative IL-1β, TNF-α, and IL-6 mRNA levels were quantified and normalized to GAPDH gene expression using two-step qRT-PCR. In the first step, purified RNA was quantified and first-strand cDNA synthesis was performed on ∼3 µg RNA in a 30 µl reaction using an Omniscript RT kit (Qiagen, Valencia, CA, USA). In the second step, IL-1β, TNF-α, IL-6, and GAPDH transcription in each sample was quantified in separate reactions on an Opticon2 DNA Engine (MJResearch, Waltham, MA, USA) using target-specific primers and a target-specific fluorescence resonance energy transfer (FRET) probe (Biosource, Camarillo, CA, USA). Primer and FRET probe sequences were as follows: IL-1β 5′-CTGTCGGACCCATATGAGC-3′ (forward), 5′-GCTCATGGAGAATATCACTTGTTG-3′ (reverse), 5′-FAM-AAGCTCTCCACCTCAATGGACAGA-BHQ1-3′ (FRET probe); TNF 5′-AGACCCTCACACTCAGATCATG-3′ (forward), 5′-TCAGCCACTCCAGCTGCT-3′ (reverse), 5′-FAM-CCACGTCGTAGCAAACCACCA-BHQ1-3′ (FRET probe); IL-6 5′-GCCCACCAAGAACGATAGTC-3′ (forward), 5′-GTGGTTGTCACCAGCATCAG-3′ (reverse), 5′-FAM-CTGCAAGAGACTTCCATCCAGTTGC-BHQ1-3′ (FRET probe); GAPDH 5′-CCTCAACTACATGGTCTACATGTTC-3′ (forward), 5′-GCTCCTGGAAGATGGTGATG-3′ (reverse), 5′-FAM-CATTCTCGGCCTTGACTGTGCCGT-BHQ1-3′ (FRET probe). Each qRT-PCR target was measured with 5 replicate wells for each sample (n = 5). Each qRT-PCR experiment was performed in at least three independent populations of macrophages.

### Analysis

For qRT-PCR analysis of IL-1β, TNF-α and IL-6 mRNA, cytokine ΔC_T_ values were compared after normalizing by subtraction of GAPDH C_T_ values from the same sample. Differences in IL-1β, TNF-α and IL-6 ΔC_T_ values between drug-treated and control cells were analyzed using a two-tailed t-test at the 95% confidence interval. Fold expression of each cytokine in drug treated relative to control cells was calculated using the ΔΔ C_T_ method. Differences between levels of IL-1β, TNF-α and IL-6 protein in macrophage culture media by group were analyzed using a two-tailed t-test at the 95% confidence interval. All data are presented as the mean +/− standard error of the mean. *P*<0.05 was considered to be statistically significant and is denoted by an asterisk.
